# Oxytocin as a peripheral biomarker for Autism Spectrum Disorder: a systematic review and meta-analysis

**DOI:** 10.1192/j.eurpsy.2022.254

**Published:** 2022-09-01

**Authors:** A. Natale, L. Fusar-Poli, S. Sturiale, C. Concerto, A. Aguglia, A. Amerio, G. Serafini, M. Amore, E. Aguglia

**Affiliations:** 1 University of Catania, Department Of Clinical And Experimental Medicine, Psychiatry Unit, Catania, Italy; 2 IRCCS Ospedale Policlinico San Martino, Genoa, Italy, Department Of Neuroscience, Rehabilitation, Ophthalmology, Genetics, Maternal And Child Health, Section Of Psychiatry, University Of Genoa, Genoa, Italy, Genoa, Italy

**Keywords:** Autism Spectrum Disorder, peripheral biomarker, Oxytocin, systematic review

## Abstract

**Introduction:**

Autism spectrum disorder (ASD) is a group of life-long neurodevelopmental conditions characterized by impairments in social communication and by the presence of restricted interests or repetitive behaviors. Several genetic, biological, and psychosocial mechanisms seem to play a role in the etiopathogenesis of this complex condition. Preclinical models have shown a potential role of oxytocin (OT), a peptide involved in a complex range of behaviors, including those related to social interaction. Therefore, it has been hypothesized that OT levels may be decreased in autistic people.

**Objectives:**

To compare the levels of peripheral OT in autistic people vs neurotypical controls.

**Methods:**

We performed a systematic literature search up to December 2020 according to PRISMA guidelines. Final inclusion was based on the following criteria: (1) Participants: individuals of any age diagnosed with ASD; (2) Controls: neurotypical subjects; (3) Outcome: OT levels, either in saliva, serum, or plasma; (4) Study design: case-control. Meta-analyses are ongoing.

**Results:**

We finally included 21 papers published between 1998 and 2020, of which one recruited adult participants. Fifteen studies measured OT levels in plasma, 4 in saliva, and 2 in serum. Preliminary meta-analyses on 10 studies showed that peripheral OT levels in autistic individuals are reduced compared to neurotypical controls, with sex differences.

**Conclusions:**

Our preliminary findings show that peripheral OT might represent a potential biomarker for ASD. Future well-conducted case-control studies with a detailed phenotypical characterization of samples are needed to understand the role of OT deficits in specific subgroups.

**Disclosure:**

No significant relationships.

